# Neural Representation of the Coding of Language and Speech in Children with Congenital Toxoplasmosis

**DOI:** 10.3390/diagnostics16213604

**Published:** 2026-11-01

**Authors:** Laís Ferreira, Milaine Dominici Sanfins, Márcia Keske-Soares, Piotr Henryk Skarzynski, Denis Altieri de Oliveira Moraes, Eliara Pinto Vieira Biaggio

**Affiliations:** 1 Department of Speech-Hearing-Language, Federal University of Santa Maria, Santa Maria 97105-900, Brazil; lais.ferreira@univali.br (L.F.); marcia-keske.soares@ufsm.br (M.K.-S.); eliara.pv.biaggio@ufsm.br (E.P.V.B.); 2 Post-Graduate Program in Human Communication Disorders, Federal University of Santa Maria, Santa Maria 97105-900, Brazil; denis.moraes@ufsm.br; 3 Department of Speech-Hearing-Language, University of Vale do Itajaí, Itajaí 88302-901, Brazil; 4 Department of Speech-Hearing-Language, Universidade Federal de São Paulo, São Paulo 04044-020, Brazil; 5 Post-Graduate Program in Clinical Audiology, Instituto de Ensino e Pesquisa Albert Einstein, São Paulo 05652-000, Brazil; 6 Department of Teleaudiology and Screening, World Hearing Center, Institute of Physiology and Pathology of Hearing, 05-830 Kajetany, Poland; p.skarzynski@ifps.org.pl; 7 Center of Hearing and Speech Medincus, 05-830 Kajetany, Poland; 8 Institute of Sensory Organs, 05-830 Warsaw, Poland; 9 Statistics Department, Federal University of Santa Maria, Santa Maria 97105-900, Brazil

**Keywords:** auditory evoked potentials, hearing, congenital toxoplasmosis

## Abstract

**Purpose**: Children diagnosed with congenital toxoplasmosis (CT) are at increased risk of central auditory nervous system dysfunction, potentially affecting their hearing skills and language development, even in the absence of direct hearing loss. This study therefore explored whether electrophysiological measures of language and speech were affected in children with CT. **Method:** This prospective longitudinal cohort study recruited 13 children diagnosed with CT (the CT group) and 25 typically developing children (control group). Participants underwent frequency-following response (FFR) recording at three developmental stages: stage 1 (S1) covering the first to third month of life; stage 2 (S2) spanning the sixth to tenth month; and finally stage 3 (S3) from 4 to 6 years, although by then losses to follow-up were severe (>50%). During S3, language and speech development assessments were also conducted. **Results**: Logistic regression between FFR components and expressive language assessments showed no significant differences, although statistical power was compromised by the small number of remaining children. There was no significant interaction between the FFR components across the stages or between groups (*p* > 0.05). Main-effect analyses revealed that children with congenital toxoplasmosis exhibited significantly prolonged latencies relative to controls for waves A (*β* = 0.38 ms, 95% CI [0.11, 0.65], *p* = 0.008), F (*β* = 0.46 ms, 95% CI [0.01, 0.91], *p* = 0.048), and O (*β* = 0.87 ms, 95% CI [0.40, 1.34], *p* < 0.001). **Conclusions**: Our findings indicate that children diagnosed with CT exhibit prolonged latencies of waves A, F, and O and reduced slope values in the FFR, suggesting impairments in neural speech sound coding from the first months of life into early childhood, independent of the assessment stage. Although FFR latencies did not predict expressive language outcomes, all children in the CT group assessed at S3 presented language and/or speech alterations, which suggests a possible association between congenital toxoplasmosis and speech development. Given the small number of children assessed at this stage, this is a preliminary finding that warrants further investigation in larger longitudinal studies.

## 1. Introduction

Congenital toxoplasmosis (CT) represents a significant public health challenge, particularly in tropical regions such as Central and South America. It is transmitted from mother to fetus via the transplacental passage of the *Toxoplasma gondii* parasite during the acute stage of maternal infection. Alongside syphilis, rubella, cytomegalovirus, and herpesvirus, CT is among the most common congenital infections and a major cause of neonatal morbidity and mortality [1,2]. Despite its prevalence, diagnosing CT poses challenges due to the lack of clinical manifestations in most pregnant women and the absence of symptoms or clinical signs in approximately 90% of infected newborns. 

Diagnosis typically relies on serological testing and clinical evaluations of the newborn [3]. Children diagnosed with CT often exhibit ophthalmological and/or neurological alterations, seen in 60–80% of cases. These can lead to severe fetal and neonatal repercussions, including changes in cranial volume, ventricular dilation, intracerebral calcifications, and convulsions [4,5,6]. Moreover, there is an appreciable risk of later sequelae, indicating that clinical signs can manifest both neonatally and during the first months of life [4,7]. 

Other consequences of CT are effects on hearing. Indeed, CT infection is a risk indicator for hearing impairment and highlights the need for targeted hearing and language testing in affected children [8]. Peripheral hearing loss, sensorineural hearing loss, and mild to moderate hearing loss have all been associated with CT [9,10,11,12,13,14,15]. Other research points to differences in the functioning of the central auditory nervous system between children with CT and those developing typically. The differences include a higher risk of alterations in brainstem tests, auditory skill behavior, and language development [14,16,17,18]. 

In this context, subcortical evaluations using the frequency-following response (FFR) often show prolongation of certain wave potentials, regardless of the child’s hearing thresholds [19]. However, the impact of CT on the encoding of complex sounds and on language development in early childhood remains understudied, and there is a need for further study into how CT affects speech coding and language aspects in young children. Thus, the aim of this study was to study how FFR measures differed between children with CT and those with typical development at three stages, from the first months of life up to 6 years. A second aspect was to explore whether a relationship could be found between the FFR findings and language and speech outcomes at preschool age as assessed by the Language Development Evaluation 2 (ADL-2) questionnaire. 

## 2. Material and Methods

### 2.1. Study Design and Ethical Aspects

This was a prospective longitudinal comparative cohort study. Participants were allocated according to exposure status (congenital toxoplasmosis or typical development) and followed prospectively, with FFR recordings at three scheduled developmental stages (S1, S2, and S3), complemented with language and speech assessments at S3. The study’s reporting follows the STROBE guidelines for cohort studies.

This study was approved by the research ethics committee (CAAE no. 81117517.0.0000.5346 and opinion no. 2.538.043). Participation was limited to individuals whose parents or guardians provided signed consent after being fully informed about the study’s procedures. This included an assent form for literate children and adolescents. All ethical principles outlined by the Brazilian National Health Council, which establishes norms and regulatory guidelines for research involving human subjects, were strictly adhered to.

### 2.2. Participants and Sample Selection Procedures

During 2018 and 2019, 37 children of both sexes were recruited from the Neonatal Hearing Screening program and the Hearing and Language Follow-up Clinic for children with CT. All participants came from a public hospital in central Rio Grande do Sul, southern Brazil, and received healthcare through the Brazilian Unified Health System (SUS), suggesting potentially similar socioeconomic backgrounds. The study population were children diagnosed during a toxoplasmosis outbreak in a small inland city of Brazil, and the sample reflected the population served by the public hospital where the study was conducted.

The children were assessed at three developmental stages (S1 to S3) beginning from the first month of life and ending at 4–6 years of age. Stage 1 (S1) occurred between the first and third month of life, stage 2 (S2) from the sixth to the tenth month, and stage 3 (S3) from 4 to 6 years of age. The assessment periods were partly determined by COVID-19 pandemic constraints.

For inclusion in the study, all participants underwent selection procedures appropriate to their age and stage of development. At S1, hearing was assessed using the neonatal hearing screening test involving bilateral brainstem auditory evoked potentials and/or otoacoustic emissions [8]. At S2, infants needed to show minimum hearing levels for their age range at frequencies of 0.5, 1, 2, and 4 kHz, determined through visual reinforcement audiometry. Finally, at S3, participants were expected to exhibit, at the same frequencies, pure tone hearing thresholds within the normal range for their age, as well as achieve a speech recognition threshold as measured by logoaudiometry [20].

Additionally, children underwent tympanometry and brainstem auditory evoked potential testing at all three stages. Tympanometry was performed using a 1 kHz probe tone in children up to 9 months of age and a 226 Hz probe tone in children older than 9 months and showed no evidence of middle ear alterations. The brainstem auditory evoked potential test was performed using a click stimulus and needed to show normal parameters appropriate for their age, including reproducible waves I, III, and V, absolute latencies, and interpeak intervals [21].

The final sample consisted of a CT group of 13 children diagnosed with CT and a control group of 25 healthy children. Children in the CT group were treated appropriately by a specialized multidisciplinary team at the hospital, adhering to official guidelines [22]. Diagnosis was based on clinical assessments and serological testing of blood samples from the newborns, supplemented with nonspecific tests such as hematology (complete blood count), cerebrospinal fluid analysis, biochemistry, skull computed tomography, or skull ultrasound (with skull X-ray for intracranial calcifications if the former were not feasible). 

An ophthalmological assessment, including a fundus examination, was conducted to identify signs of uveitis and retinochoroiditis. Confirmation of congenital infection relied on detecting the newborn’s IgM and/or IgA antibodies, with diagnosis of CT confirmed by persistent specific IgG antibodies at 12 months of age. Treatment commenced from birth and consisted of using sulfadiazine, pyrimethamine, and folinic acid for 1 year.

The control group comprised healthy, term-born children (gestational age between 37 and 42 weeks) with an Apgar score greater than 8 at the 1st and 5th minutes of life, birth weight greater than 1500 g, general health deemed adequate based on hospital records, and satisfactory overall development based on regular childcare follow-up in the early years. Table 1 lists the characteristics of the groups at each stage of evaluation. 

### 2.3. Research Procedures

#### 2.3.1. Frequency-Following Response Measurements

FFRs were assessed at each of the three developmental stages for the control and CT groups and measured using the Intelligent Hearing Systems SmartEP device. The child’s skin was cleaned with an abrasive gel and surface electrodes positioned at Fz, Fpz, M1, and M2 [23]. Electrode impedance was maintained below 3 kΩ.

FFRs were measured using a/da/syllable of 40 ms delivered monaurally at 80 dBnHL to the right ear. Stimuli were played through ER-3A insert earphones at 10.9 stimuli per second, with alternating polarity and an analysis window of 80 ms. A band-pass filter of 0.1–3 kHz was used. All children received two sweeps of 3000 stimuli each, and these were averaged to produce the resultant wave. The key components of this potential (V, A, C, D, E, F, and O) were identified by two professionals experienced in auditory electrophysiology; they were blinded to which group the participant had been allocated (CT or control).

Infants up to 3 months old were assessed during natural sleep, positioned comfortably in their caregiver’s lap. Infants older than 6 months were awake and seated in their caregiver’s lap during the assessment, while children over 3 years were evaluated while awake and calmly seated in a recliner. The average duration of data collection was 30 min. Examinations with artifact levels exceeding 5% were excluded, and a new recording attempt was made.

#### 2.3.2. Language and Speech Assessments

As part of S3, a speech therapist conducted speech and language assessments. They were blinded to the participant’s group (congenital toxoplasmosis or control) and to their FFR results. The Language Development Evaluation 2 (ADL-2) tool was used to evaluate the levels of both receptive and expressive language, including its content (semantics) and structure (morphology, phonology, and syntax) [24]. Receptive and expressive language were evaluated separately, centering on the child’s phonological, lexical/semantic, morphosyntactic, phonological awareness, memory, and inferencing abilities. ADL-2, which is designed to measure receptive and expressive language in children aged from 3 years to 6 years 11 months, was selected because of its applicability to the Brazilian child population and was applied to both groups in the study. ADL-2 provides age-specific scores for receptive and expressive language as well as a global language score (GLS) derived by adding the two scores.

The GLS was used to classify the children’s language development based on the following criteria: a score of 115–85 was considered normal, 84–77 indicated a mild language disorder, 76–70 indicated a moderate language disorder, and ≤69 indicated severe language disorder. 

Comprehensive vocabulary, a facet of comprehensive language, was evaluated using the receptive vocabulary test [25]. This tool is based on 33 pictorial items, each accompanied by a target word and four distractors. The child is required to identify the image that matches the examiner’s spoken word. Scoring is based on the number of correct answers, with raw scores ranging from 0 to 33 points. The scores are then compared to normative data to classify the vocabulary level as very low, low, average, high, or very high. For this study, the latter three were deemed ‘adequate,’ whereas the first two were considered ‘altered.’ 

Similarly, expressive vocabulary and access to long-term memory were assessed using the child naming test [26], which examines the child’s ability to verbally identify 60 figures representing various objects, animals, and people. Scores were calculated similarly, with the scale ranging from very low (<70 points) to very high (≥130 points). Medium, high, and very high scores were deemed ‘adequate,’ while low and very low scores were considered ‘altered.’

Phonological assessments also included the phonological assessment tool (INFONO) [27], which tests the production of 19 Brazilian Portuguese consonants in various syllables and words. In this test, the child viewed the item on a computer screen and, in response to the examiner’s question “What is that?”, needed to name the item. Software then automatically analyzed the speech, categorizing the phonemes as correct (80–100%), partially acquired (40–79%), or absent/not acquired (0–39%) [28]. 

Lastly, the severity of speech sound disorder (SSD) was determined using the percentage of consonants correct (PCC) [29], with SSDs classified as severe (<50%), moderate–severe (50–65%), mild–moderate (65–85%), and mild (85–100%).

### 2.4. Potential Sources of Bias

Several strategies were adopted to minimize bias. To reduce selection bias, both groups were recruited from the same public hospital, through the same Neonatal Hearing Screening program and follow-up clinic, and all participants received healthcare through the Brazilian Unified Health System, which favors comparability in terms of healthcare access and socioeconomic background. To avoid confounding by peripheral hearing status, normal hearing and middle ear function were required at every stage, so that differences in FFR could not be attributed to peripheral hearing loss. To reduce measurement bias, all FFR recordings followed the same protocol and equipment at the three stages, and the FFR components were identified by two experienced examiners blinded to group allocation; likewise, the speech therapist who conducted the language and speech assessments was blinded to group allocation. Attrition bias was a recognized concern, given the loss to follow-up across stages, partly due to the COVID-19 pandemic. To address it, linear mixed-effects models were used, which incorporate all available observations from each participant without excluding those with incomplete follow-up, under the assumption that data were missing at random.

### 2.5. Data Analysis

The following time-domain variables were extracted and tabulated: absolute latencies of waves V, A, C, D, E, F, O, and slope measurements in the FFR. The A–D and A–O interpeak intervals were calculated as the difference between the absolute latencies of the respective waves, and the slope of the V–A complex (µV/ms) was calculated as the amplitude difference between waves V and A divided by the latency difference between them. Statistical analyses were conducted using R and SPSS software (version 22) (R Core Team: Vienna, Austria, 2023; IBM Corp.: New York, NY, USA, 2013). We also tested for normality (Shapiro–Wilk), homogeneity of variances between groups (Levene’s test), and the adequacy of the models (significance of coefficients and normality of residuals). 

Linear mixed-effects models were fitted, including fixed effects for group, developmental stage, and their interaction, with random intercepts specified for subjects. Taking into account sample attrition over the course of the study, the precision of our inferences is captured by the 95% confidence intervals of the estimated model coefficients. 

When assessing the normality of language-related variables by group, only the variables from the naming test met this criterion (*p* = 0.537; *p* = 0.091) and were thus analyzed using a parametric *t*-test for independent groups; other variables were compared using Mann–Whitney and Wilcoxon tests. Binary logistic regression models were applied to explore the association between group and language variables. Additionally, we examined the relationship between language variables from the ADL-2 test and all components of the FFR using logistic models. 

## 3. Results

No significant Group × Developmental Stage interactions were detected across the evaluated FFR measures (*p* > 0.05); final models were therefore specified with main effects only. Given the limited sample size, however, the absence of interaction should not be construed as definitive evidence of independence between group and developmental effects. Main-effect analyses revealed that children with congenital toxoplasmosis exhibited significantly prolonged latencies relative to controls for waves A (*β* = 0.38 ms, 95% CI [0.11, 0.65], *p* = 0.008), F (*β* = 0.46 ms, 95% CI [0.01, 0.91], *p* = 0.048), and O (*β* = 0.87 ms, 95% CI [0.40, 1.34], *p* < 0.001). Conversely, no group-level differences emerged for waves V, C, D, or E, nor for the A–D and A–O interpeak intervals. 

In terms of developmental progression, S2 was characterized by shorter latencies relative to S1 across waves V (*β* = −0.64 ms, *p* < 0.001), A (*β* = −0.65 ms, *p* < 0.001), D (*β* = −0.83 ms, *p* = 0.001), E (*β* = −0.71 ms, *p* = 0.007), F (*β* = −0.79 ms, *p* = 0.001), and O (*β* = −0.84 ms, *p* = 0.003). At S3, latencies remained significantly shortened relative to baseline for waves V (*β* = −0.58 ms, *p* < 0.001), A (*β* = −0.62 ms, *p* < 0.001), and D (*β* = −0.76 ms, *p* = 0.004), whereas the A–O interval demonstrated significant prolongation (*β* = 0.91 ms, 95% CI [0.29, 1.54], *p* = 0.005). Altogether, these longitudinal trajectories reflect age-related neural maturation in specific FFR components, whereas group impairments were restricted to waves A, F, and O (Table 2).

**Figure 1:**
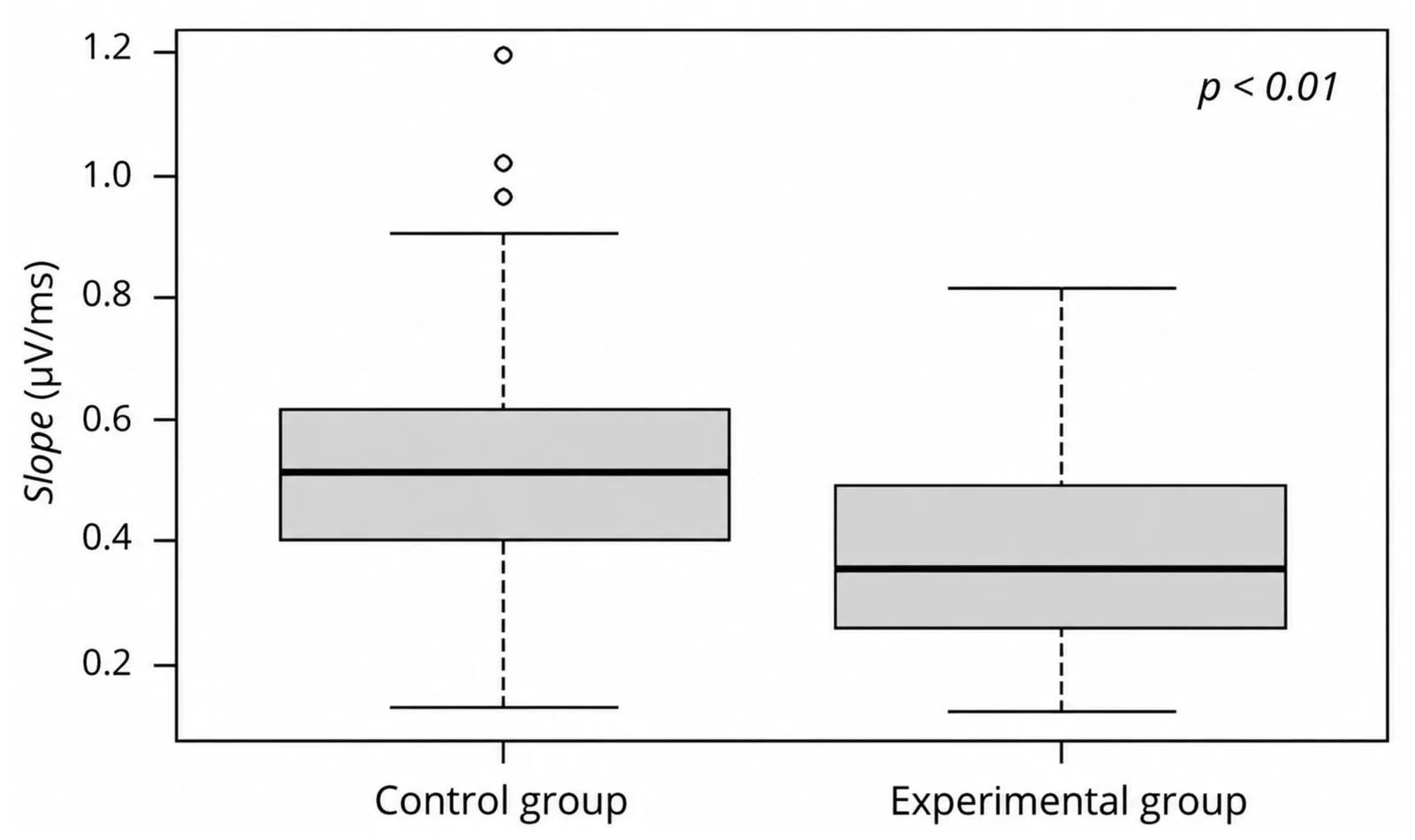
Significantly lower slope measures in the CT group compared to the control group (*p* < 0.01).

For the slope measurement (µV/ms), there was no interaction (*p* = 0.120) between the group and stages; however, there was a significant difference between groups (*p* < 0.010) (Figure 1), with significantly lower values in the CT group. This result suggests that CT may affect speech coding in early childhood, either soon after birth or over the following few years. 

Turning now to ADL-2 scores, the analysis here was hampered by a severe loss to follow-up in both the control group and the experimental group (see Table 1). This meant there was low statistical power in efforts to correlate FFR results and ADL scores. There is no doubt that small *n*-values were a major factor behind our finding that no significant difference (*p* > 0.05) was found between the groups for any component when our logistic regression model of FFR components and expressive ADL-2 scores was run. 

Nevertheless, within the CT group, we found that all the children had altered language and/or speech at S3. Of the 7 children remaining, 2 of them had a language disorder with reduced receptive and expressive vocabulary and severe SSD, while another child had altered expressive language and reduced receptive vocabulary, with mild–moderate SSD. Of the other 4 children, 3 had mild–moderate SSD and 1 had moderate–severe SSD. 

In contrast, however, for the control group descriptive measures from the language assessments showed that none of the 6 remaining children exhibited significant changes in the ADL-2 assessments of comprehensive or expressive language, even though 2 of them were still acquiring phonemes.

## 4. Discussion

This study adds to the growing body of evidence that children with CT exhibit functional impairments in the neural coding of speech sounds despite having normal hearing thresholds. Specifically, we observed some delays in processing speech sounds in this population, indicating that acoustic information may be encoded more slowly in children with CT compared to their healthy peers. The speech and language assessments in S3 show that this condition has an impact on speech coding and language aspects during early childhood. 

Understanding speech sounds involves perceiving and encoding both the temporal and spectral information in consonants and vowels. This process requires processing the acoustic characteristics of sounds—such as frequency, intensity, and duration—and identifying linguistic patterns. Therefore, the functional integrity of the neural mechanisms responsible for speech coding is essential for interpreting messages [30,31]. 

Our study employed the syllable /da/ to elicit electrophysiological responses linked to the speech sound coding process. The results indicate that the CT group exhibited prolonged absolute latencies for waves A, F, and O of the FFR and decreased slope measurements. This suggests that the observed functional impairments in children with CT may affect both the coding of the consonant /d/ and the vowel /a/, highlighting difficulties in processing both rapid and short sounds and sounds with periodic and harmonic structures. These findings underscore the importance of audiological and language monitoring for children with CT early on, as such impairments can hinder language and speech acquisition and development [32,33].

The V and A components, representing the transient portion of the response, provide insight into the coding of rapid temporal changes. These components capture the abrupt transitions in frequency and intensity of acoustic stimuli, illustrating the neural processing of coding consonants [34,35]. Given the prolongation observed in wave A, it may be hypothesized that children with CT experience difficulties in encoding short-duration sounds. In contrast, peak C, which conveys neural activity data related to the transition between the consonant /d/ and the vowel /a/, did not differ significantly between the groups. 

Among the components related to the vowel, only wave F showed significant prolongation, whereas waves D and E did not differ between groups. These components reveal information about the coding of periodic and harmonic sound structures of the vowel, such as the fundamental frequency and formants [34,35]. Furthermore, the O component—also known as the “offset response” and crucial for the temporal perception of speech—was influenced by CT, showing prolongation throughout early childhood development in the CT group [35].

Differences in the absolute latency of waves A, F, and O were observed alongside lower slope values in children with CT. The slope component, which is related to the temporal synchronization of response generators, is quantified using the absolute latency and amplitude values of the V–A complex [34]. Prior studies have reported alterations in slope measurements in children with learning and language disorders [30,36], highlighting the significance of this component in child development. Our study found reduced slope values in the CT group, with no evidence of stage-dependent differences, suggesting that children diagnosed with CT may exhibit deficits in coding complex sounds throughout early childhood. This evidence underscores the need for specialized monitoring of expressive and comprehensive language in children with CT, as recommended by national and international guidelines [8,37,38]. 

Language assessment conducted during the third stage showed that latency values of FFR components were not associated with expressive language outcomes in this sample; given the small number of children assessed at S3, this finding should be interpreted with caution. This assessment aids in identifying issues in language content and structure acquisition and development in preschool-aged children. 

Although FFR latencies did not predict expressive language outcomes, all children in the CT group assessed at S3 showed language and/or speech alterations, which raises the hypothesis that early impairments in coding complex sounds may be related to later language and speech difficulties; this hypothesis requires confirmation in larger samples. These results align with prior research indicating that abnormalities in FFRs are indicative of difficulties in detecting speech sound cues and coding, which is crucial for language acquisition [39]. Speech comprehension relies on interpreting rapid changes in temporal and spectral information within consonants and vowels, suggesting that speech perception difficulties can originate from a deficit in speech sound coding [30].

The observed deficits in speech coding assessment highlight the importance of early language acquisition, development, and comprehension processes, which later influence additional linguistic modalities. Previous research has noted that pitch processing and neural coding mechanisms undergo maturation in the brainstem early in life [40,41]. Therefore, the prolonged FFR wave delays and reduced slope measurements observed up to the age of 4–6 years may represent a risk for language and speech development.

Other researchers have proposed the FFR as a potential tool for identifying children at risk for language and speech disorders [32,33,34,39]. Although our data did not confirm a predictive relationship, the group differences observed are consistent with this perspective and warrant investigation in larger cohorts. By identifying deficits in complex sound coding in early years, FFR may support monitoring and early intervention for children at risk of developing language, speech disorders, and subsequent educational challenges.

From this perspective, several authors have suggested that the early identification of prolonged FFR waves offers valuable data for clinical settings, particularly because younger infants are more likely to remain asleep or quiet during assessments. This facilitates easier evaluation and early diagnosis. Importantly, FFR represents a screening alternative for auditory processing, primarily occurring in the midbrain. It reflects auditory nerve, brainstem, and cortical contributions in infants at risk of developing language disorders [41,42], complementing newborn hearing screening programs. Furthermore, this aligns with evidence indicating that CT can adversely affect child development [14,17,18,43]. This underscores the social and public health impact of this condition, which is relevant for programs such as the Brazilian Unified Health System and in the private sector. Moreover, monitoring children with CT in hearing health programs should extend beyond peripheral hearing assessment to also include the central auditory nervous system functions and language and speech aspects, necessitating health professional involvement.

The sample size limitations, particularly the reduction in participant numbers over the study period, reflect the challenges researchers in Brazil face, including the costs associated with longitudinal child health and development research. The COVID-19 pandemic further affected longitudinal follow-up and participant attendance in various study stages. Regarding the potential effects of the COVID-19 pandemic on children’s language development, social isolation and reduced social interaction may have affected both groups. Therefore, these factors were not exclusive to either group and should be considered when interpreting the findings.

Nevertheless, the observed results encourage further longitudinal research across different populations to understand better how FFR contributes to speech sound processing alterations. This facilitates expanding research into the phonetic and phonological inventory changes and speech sound discrimination (auditory speech perception assessments) in these children. Such insights, paired with FFR findings, can assist clinicians in planning and implementing effective language rehabilitation strategies.

This electrophysiological response provides a novel approach to exploring speech neural processing, which is essential for language acquisition. Specifically, the FFR enables the analysis of the temporal and spectral characteristics of speech, which is crucial for the interaction between hearing and language [39,44]. 

Some limitations should be considered when interpreting the findings of this study. These include the reduction in sample size over the study period, particularly at S3, which limits the conclusions that can be drawn regarding language assessment outcomes, and the limited characterization of the clinical and medical features of the group of children with congenital toxoplasmosis. These aspects should be addressed in future studies to support more comprehensive interpretations and strengthen the conclusions regarding the developmental outcomes investigated. Although mixed-effects models make use of all available data, losses to follow-up may not have been random; if children with greater difficulties were less likely to return, the differences between groups at S3 may have been underestimated or overestimated.

## 5. Conclusions

Our findings indicate that children diagnosed with CT exhibit prolonged latencies of waves A, F, and O and reduced slope values in the FFR, suggesting impairments in neural speech sound coding from the first months of life into early childhood, independent of the assessment stage. Although FFR latencies did not predict expressive language outcomes, all children in the CT group assessed at S3 presented language and/or speech alterations, which suggests a possible association between congenital toxoplasmosis and speech development. Given the small number of children assessed at this stage, this is a preliminary finding that warrants further investigation in larger longitudinal studies.

## Figures and Tables

**Table 1:** Number of subjects (*n*) at each stage of the study, together with the number having associated comorbidities.

Group	*n*	*n* (f)	*n* (m)	*n* (S1)	*n* (S2)	*n* (S3)	Congenital Toxoplasmosis + Chorioretinitis
CT group	13	6	7	13	11	7	4
Control	25	16	9	25	8	6	0

S1: stage 1 (between the 1st and 3rd month of life); S2: stage 2 (6th–10th month of life); S3: stage 3 (4–6 years of age); f: female; m: male.

**Table 2:** Absolute latencies of FFR waves by group and developmental stage.

Response (Latencies)	Experimental Group			Control Group			Group Effect			S2 vs. S1			S3 vs. S1		
	S1	S2	S3	S1	S2	S3	β	IC95%	*p*	β	IC95%	*p*	β	IC95%	*p*
V	7.7	7.0	7.8	7.5	6.8	6.8	0.22	−0.00–0.44	0.052	−0.64	−0.78–−0.50	<0.001	−0.58	−0.73–−0.42	<0.001
A	8.8	8.2	9.0	8.5	7.7	7.7	0.38	0.11–0.65	0.008	−0.65	−0.85–−0.44	<0.001	−0.62	−0.85–−0.39	<0.001
C	18.1	18.1	19.3	17.9	17.0	17.6	0.38	−0.28–1.04	0.250	−0.24	−0.67–0.18	0.257	0.14	−0.34–0.61	0.571
D	24.2	23.4	24.1	23.9	23.0	23.0	0.36	−0.07–0.78	0.095	−0.83	−1.28–−0.37	0.001	−0.76	−1.27–−0.26	0.004
E	32.5	31.7	33.4	32.1	31.4	31.6	0.47	−0.09–1.04	0.094	−0.71	−1.20–−0.21	0.007	−0.06	−0.62–0.49	0.816
F	40.9	40.0	41.3	40.4	39.7	40.1	0.46	0.01–0.91	0.048	−0.79	−1.24–−0.35	0.001	−0.22	−0.71–0.27	0.370
O	49.5	48.8	50.2	48.8	47.8	48.9	0.87	0.40–1.34	<0.001	−0.84	−1.40–−0.29	0.003	0.24	−0.37–0.85	0.433
A–D	15.4	15.2	15.2	15.4	15.3	15.3	−0.04	−0.51–0.42	0.848	−0.13	−0.60–0.34	0.585	−0.10	−0.63–0.42	0.689
A–O	40.7	40.6	41.5	40.2	40.1	41.2	0.45	−0.04–0.93	0.071	−0.12	−0.69–0.45	0.678	0.91	0.29–1.54	0.005

S1: stage 1 (1st–3rd month of life); S2: stage 2 (6th–10th month of life); S3: stage 3 (4–6 years of age); *β*: model estimated coefficient; IC: confidence interval; *p*: exact level of significance.

## Data Availability

The data supporting the findings of this study are not publicly available. However, further information may be obtained from the corresponding author upon reasonable request.
